# Thanatomicrobiome composition profiling as a tool for forensic investigation

**DOI:** 10.1080/20961790.2018.1466430

**Published:** 2018-05-31

**Authors:** Wei Zhou, Yingnan Bian

**Affiliations:** a The Jackson Laboratory for Genomic Medicine, Farmington, CT, USA; b Shanghai Key Laboratory of Forensic Medicine, Shanghai Forensic Service Platform, Academy of Forensic Science, Shanghai, China

**Keywords:** Forensic science, forensic genetics, thanatomicrobiome, microbiome, postmortem microbiome, PMI, prediction model

## Abstract

Thanatomicrobiome, or the postmortem microbiome, has been recognized as a useful microbial marker of the time and location of host death. In this mini-review, we compare the experimental methods commonly applied to thanatomicrobiome studies to the state-of-the-art methodologies in the microbiome field. Then, we review present findings in thanatomicrobiome studies, focusing on the diversity of the thanatomicrobiome composition and prediction models that have been proposed. Finally, we discuss potential improvements and future directions of the field.

## Introduction

The human microbiome project has revealed that there are 10 times more microbial cells than human cells in and on the surface of a human body [1,2]. The spatial temporal diversities of the microbes are surprisingly large: not all microbes are present on all body sites [1–4], certain microbes and microbial assemblages colonize of certain body parts, and the composition of the microbial communities change due to intrinsic (that is, health and disease states of the host) and extrinsic (for example, temperature [5,6], humidity [6], use of drugs and antibiotics [7] and diet [8]) factors. These changes, albeit dynamic, are often highly predictable [5,6,8,9]. It is not only intriguing how the microbiome mechanistically interacts with the intrinsic and extrinsic factors, but also of great interest is its potential to serve as a microbial marker of these factors.

The concept of the microbiome composition acting as a biomarker has also been applied after the host has died, and is especially relevant in the field of forensic sciences. The microbial community associated with the host after death is referred to as thanatomicrobiome [10], named after Thanatos, the representation of death in Greek mythology. Upon death, host tissues and cells decompose and cellular components are released to the surrounding tissues, resulting in significant changes in the host environment overtime [11–13]. Such changes shape, and are influenced by, host microbes as well as environmental microbes, resulting in characteristic microbial community dynamics specific to thanatomicrobiome. Although thanatomicrobiome is a relatively new topic in forensic sciences and microbiome studies, its potential as an addition to the forensic toolbox is enormous. In this paper, we review present studies of thanatomicrobiome in two aspects. First, we review the methods that have been used to profile thanatomicrobiome and compare them to the state-of-the-art methods applied in other microbiome studies. Next, we review major findings in the community characteristics of thanatomicrobiome, with a focus on the diversity and robustness of the microbial communities, proposed microbial markers, and the usefulness of (or the lack of) proposed predictive models.

## Methods in microbial community profiling and their applications in thanatomicrobiome studies

Most microbiome studies involve generating compositional profiles of the microbiome, through characterizing what microbes are present and estimating their abundance – both of which are strongly associated with the spatial–temporal traits of the community [1,2,14]. Accurate compositional profiling of thanatomicrobiome is the first step towards identifying forensically relevant microbial markers. Microbiome composition can be investigated using culture-dependent or culture-independent method. Culture-dependent method relies on isolating or culturing living microbes from environmental samples and characterizing the cultured microbes. It allows identification of microbes with high-confidence and high-taxonomic resolution. However, a major disadvantage of such method is the bias imposed by the culture conditions [15–18]. For example, it had been estimated in the past that over 99% of the microbes in the environment cannot be cultured in a standard lab setting and over 50% of the human-associated microbes are “unculturable” [18]. Presently, new attempts of “culturomics” studies have significantly improved the diversity of culturing conditions, leading to the identification of numerous previously uncultured bacteria [19–21]. Nonetheless, being able to culture a microbe does not eliminate the culturing bias: because microbes have different ability to grow under different culture conditions, it is still extremely hard, if not impossible, to infer the abundances of the cultured microbes in their native community.

On the other hand, culture-independent method directly extract and sequence genetic materials from samples, representing a more realistic, comprehensive and high-throughput profiling of the community of interest [22–24]. Common approaches to conduct the sequencing fall in the category of marker gene-based sequencing and shotgun metagenomic whole genome sequencing (mWGS). Marker gene sequencing is conducted by first amplifying a genomic region that is conserved within a taxon but variable among taxa. Common marker genes include the 16S RNA gene for bacteria and archaea and internal transcribed spacer for fungi. Being the most commonly used culture-independent method, marker gene sequencing has the advantage of costing less time and money, but its resolution is limited by the length of the marker [25] – based on present computational methods, 16S RNA data can only generate composition-based profiling that has no higher than genus-level resolution. Even at genus-level, the method has poor discriminatory power for some taxa [26,27]. Other technical issues with 16S RNA sequencing includes isolate purity, PCR primer selection bias and possible chimeric molecule formation [28–31]. Finally, sequencing of only the marker gene region limits the investigation of functional traits of the community.

Alternatively, mWGS directly sequences all DNA material extracted from a sample. Compared to marker gene sequencing, mWGS explores a much larger sequence space and genetic diversity. Therefore, the method is naturally more costly than marker gene sequencing, but is able to profile microbial communities with much higher taxonomic resolution and is less biased [25]. Recently, it has been shown that mWGS data not only enable species-level profiling of a microbial community, but also accurate tracking of strain-level transmissions and evolutions in microbial communities [32,33]. Another advantage of mWGS is that it provides a full picture of the community – not only from a perspective of taxonomic composition, but also regarding what functional genes are present in the microbes – for all genetic sequences present in the community will be sequenced given adequate coverage. However, the major challenge for mWGS method lies in the development and choice of efficient yet accurate computational methodology to analyze the massive data [34,35]. For taxonomic profiling alone, there are dozens of software packages that classify sequencing reads based on different algorithms including sequence alignment [36,37], Bayesian classifier [38,39] or *k*-mer mapping [40,41], each of which has its advantages and limitations. On top of that, analyses results of mWGS data greatly depend on the comprehensiveness and goodness of annotation of the reference database, making cross-validation of mWGS-based findings highly difficult.

Present studies of thanatomicrobiome are predominantly based on 16S RNA sequencing. Considering the fact that 16S RNA sequencing is suitable for large number of samples, is well established for longitudinal analyses [25], provides consistent estimates, is less costly and takes less time to analyse, the method is suitable for screening relevant microbial markers to infer the time and location of death, which could be time-sensitive in forensic applications. On the other hand, the ability of mWGS to track microbial strains that transfer between samples is highly desirable in forensic science. Especially, strain-tracking is based on single nucleotide polymorphism (SNP)-resolution sequence types; its high accuracy and low probability of false positives allow the technique to serve as potential evidence of physical contact, instead of simply inferring the time and location of death. Accurate strain-tracking based on mWGS data has been successfully applied to studies on the population structure of skin microbiome [42] and vertical transmission of microbiome from mother to infants [43]. Nonetheless, application of strain tracking in a forensic setting has not yet been well established and could potentially require improvements in the efficiency of analyses methodologies.

## Community characteristics of thanatomicrobiome

The postmortem microbiome has two components: the microbial community associated with internal organs and the community associated with external body surfaces, commonly referred to as thanatomicrobiome and epinecroticmicrobiome, respectively [12]. In this section, we will use thanatomicrobiome to refer to the microbial community associated with internal organs while epinecroticmicrobiome for the community on the body surfaces; in other sections of the paper, “thanatomicrobiome” indicates the overall postmortem microbiome.

It has been well established that postmortem interval (PMI) can be inferred based on ecological succession patterns of small organisms on the cadaver. For example, the succession of carrion insect species has been used to indicate PMI [44]. However, complex organisms are often unavailable during particular weather or season [45] and have variable developmental rates [46] and oviposition time [47,48], posing limitations in practices of PMI estimation. Therefore, patterns of succession of microbes have emerged as an alternative to higher organisms. Ideally, to predict PMI based on microbial composition, a universally applicable model should be proposed that incorporates temporal variation in microbial abundance, but also has the ability to adjust for other sources of variation such as host characteristics and environments. In practice, such model is extremely hard to establish due to the limited amount of available data and the limited domain of generality of the study systems.

At present, many studies have used animal models such as rodents and swine [49–51], others used donated human bodies or bodies from criminal cases [11,52–55]. The main concern of using animal models is whether the system is similar enough to human bodies, although it has been shown that swine is similar to human in terms of the body decomposition process [56]. On the other hand, animal models are also suitable to study the fundamental mechanisms of the decomposition process so as to guide studies on human bodies, which naturally come in a much more limited number and may lead to insufficient statistical power given a significance level [12].

Upon death of the host, certain types of microbes expand in internal organs and others quiescence or go to extinct [57,58]. This change happens both in internal organs that are colonized by microbes when the host is living (such as lungs and gut), and internal sites that are sterile in a living host (such as blood, liver, spleen, heart and brain [59]) but become colonized by microbes in a sequential manner after the death of the host [13]. For non-sterile sites such as the gut, microbial richness increases after death while diversity decreases [54]. A consistently observed pattern in thanatomicrobiome succession is the continuous decline in the abundance of anaerobic taxa such as *Bacteroidetes* and *Lactobacillus*, that are abundant in the microbiome of living humans [2]. The decline of these anaerobic taxa is potentially due to the release of oxidative gases during autolysis. A large scale study confirmed the observation, and found that the pattern of thanatomicrobiome succession is similar within each sex, but dissimilar between males and females [13]. Bacteria from the order *Clostridiales* are abundant in both female and male cadavers, while *Pseudomonas* and *Streptococcus* are abundant only in female and male cadavers, respectively [13]. In terms of body sites, brain, heart, liver and spleen have the most stable community composition over time [13]. In addition, the diversity of community composition among organ tissues appears to be lower than that among individuals [10]. Quantitative predictive models are yet to be proposed for thanatomicrobiome, but it has been shown that several genera from *Firmicutes* do exhibit time signal.

Thanatomicrobiome is highly dynamic: substantial variation has been observed at the same body site at different stages of decomposition or within different phases of the same stage [53]. In addition to temporal changes at a body site, the exchange of microbes among body sites as well as between the environment and the cadaver is also highly active. Previous studies have characterized major qualitative changes in the composition of the thanatomicrobiome and assessed the potential to quantitatively associate these changes to PMI prediction. In one study, the migration of microbes from body sites into body fluid and the subsequent translocation of these microbes were observed in at least 21 bacterial genera [60]. Most recently, it has been demonstrated that *Firmicutes* sharply rise in abundance in the soil around the cadaver [11] because bacteria in the body are transferred to the soil, similar to what has been observed for the thanatomicrobiome. In addition, fly-associated taxa such as *Ignatzschineria* and *Wohlfahrtiimonas* have been shown to expand in postmortem gut microbiome [54], suggesting for the importance of environment colonization in thanatomicrobiome succession. These findings could be a useful cross-validation to thanatomicrobiome succession as an indicator for PMI estimates. Additionally, environmental microbial communities vary significantly across geographic locations and environmental types [61], therefore environmental strains identified in the thanatomicrobiome can potentially infer the location of the cadaver.

The epinecroticmicrobiome is another important biomarker of not only the time of death but also the location of death, because epinecrotic microbial communities are exposed on the surface of the body and are in close interaction with the environments, including environmental microbes. Therefore, studies on epinecroticmicrobiome usually focus on specific classes of environmental conditions. Presently, epinecroticmicrobiome has been investigated under conditions such as submersion in water [62] and placement in forests [63]. A consistent finding of these studies is that the environmental factors, such as seasons, play a vital part on the epinecroticmicrobiome [12,64,65]. Nonetheless, many of these studies have low taxonomic resolution (i.e. phylum) thus the findings could be scale-specific; high-resolution profiling of the microbiomes will allow higher statistical power, and might discover new patterns in these communities. Quantitatively, a statistical prediction model has been proposed to accurately infer the time since placement of the remains in the field [66]. The statistical prediction model was predominantly based on familial level signatures of microbial communities at the time of placement. Moreover, a machine learning model has been proposed most recently to estimate PMI based on skin microbiome composition [67].

In addition to microbiome changes on body surfaces and associated with internal organs, there are also substantial microbial changes in the substrate around the carcass, with soil being the most well-studied substrate. According to the observation of Cobaugh et al. [68], changes of soil microbial communities around human cadavers include distinct alteration in community composition (from a community abundant in *Firmicutes* and *Proteobacteria* to a community dominated by *Firmicutes*), but not overall species abundances [68]. Quantitatively, the growth curve of *Firmicutes* in soil samples around human cadavers were shown to be able to predict PMI during Tennessee summer conditions [11], although the generality of the predictive model requires further studies.

## Perspectives

Information contained in the thanatomicrobiome not only has potential to improve estimates of PMI, but could also provide clues for the decomposition location of the cadaver, both of which are of great interest to forensic investigations. In the past decade, the composition of thanatomicrobiome has been studied for its temporal patterns, environmental interactions, body site diversities and individual specificities. Many signature taxa have been proposed that could serve as microbial clocks or biomarkers of temporal and spatial traits of a given cadaver. In some cases, predictive models of PMI have been proposed.

There has not yet been a consensus method for thanatomicrobiome information collection (such as which tissues to sample, what sequencing libraries to make and what sequencing platform to use) and prediction (such as what models to use and what parameter values to include). This is largely due to the diverse and dynamic nature in the thanatomicrobiome composition collected from different hosts, environments and time. Therefore, a predictive model could be very accurate under certain conditions while not generalizable to other conditions. Such conditionally accurate models require input of prior knowledge of a given case. Another complicating factor is the taxonomic resolution at which a model should be fitted. Not surprisingly, models based on higher-resolution taxonomic profiles better explain the observed data [13] and significant within-clade variation has been suggested [55], but potential risks of over-fitting and mistakenly assigned taxonomic labels are yet to be addressed.

The succession of microbes after the death of the host is a complex process, and many aspects of thanatomicrobiome could be used to give independent estimates of the same forensic variable. These independent estimates could be combined to improve the accuracy of the forensic variable estimate using, for example, a Bayesian framework. On a broader sense, such statistical frameworks can incorporate different observations from postmortem examinations, including external and internal autopsies, molecular tests, biochemical tests and thanatomicrobiome compositions, to give more accurate insights into the time and the location of death.

## Conclusion

Thanatomicrobiome is a new yet fast developing field that links microbial community profiling techniques with forensic applications. Community dynamics of thanatomicrobiome can provide clues on decomposition location and PMI of cadavers, although interpretation of the dynamics is complicated by environmental and technical factors, and a quantitative predictive model could have application only in certain scenario types. Future implementation of state-of-the-art profiling techniques such as species- and strain-resolution metagenomics in thanatomicrobiome studies could provide new insights into spatial temporal features of forensic samples.
